# Hunger, Food Cravings, and Diet Satisfaction are Related to Changes in Body Weight During a 6-Month Behavioral Weight Loss Intervention: The Beef WISE Study

**DOI:** 10.3390/nu10060700

**Published:** 2018-05-31

**Authors:** R. Drew Sayer, John C. Peters, Zhaoxing Pan, Holly R. Wyatt, James O. Hill

**Affiliations:** 1 University of Colorado Anschutz Health and Wellness Center, University of Colorado Anschutz Medical Campus, Aurora, CO 80045, USA; John.C.Peters@ucdenver.edu (J.C.P.); Holly.Wyatt@ucdenver.edu (H.R.W.); James.Hill@ucdenver.edu (J.O.H.); 2 Department of Pediatrics, Children’s Hospital Colorado Research Institute, Anschutz Medical Campus, Aurora, CO 80045, USA; Zhaoxing.Pan@ucdenver.edu

**Keywords:** weight loss, dietary protein, appetite, cravings, dietary adherence, diet satisfaction

## Abstract

Previously published findings from the Beef WISE Study (Beef’s Role in Weight Improvement, Satisfaction, and Energy) indicated equivalent weight loss between two energy-restricted higher protein (HP) diets: A HP diet with ≥4 weekly servings of lean beef (B; *n* = 60) and a HP diet restricted in all red meats (NB; *n* = 60). Long-term adherence to dietary prescriptions is critical for weight management but may be adversely affected by changes in appetite, food cravings, and diet satisfaction that often accompany weight loss. A secondary *a priori* aim of the Beef WISE Study was to compare subjective ratings of appetite (hunger and fullness), food cravings, and diet satisfaction (compliance, satisfaction, and deprivation) between the diets and determine whether these factors influenced weight loss. Subjective appetite, food cravings, and diet satisfaction ratings were collected throughout the intervention, and body weight was measured at the baseline, after the weight loss intervention (week 16), and after an eight-week follow-up period (week 24). Hunger and cravings were reduced during weight loss compared to the baseline, while fullness was not different from the baseline. The reduction in cravings was greater for B vs. NB at week 16 only. Higher deprivation ratings during weight loss were reported in NB vs. B at weeks 16 and 24, but participants in both groups reported high levels of compliance and diet satisfaction with no difference between groups. Independent of group assignment, higher baseline hunger and cravings were associated with less weight loss, and greater diet compliance, diet satisfaction, and lower feelings of deprivation were associated with greater weight loss. Strategies to promote reduced feelings of hunger, cravings, and deprivation may increase adherence to dietary prescriptions and improve behavioral weight loss outcomes.

## 1. Introduction

Many diet options are available and effective for weight loss, but long-term adherence to dietary prescriptions and continued weight loss maintenance represent major challenges for obesity treatment [1]. Numerous studies have compared dietary patterns of differing compositions for weight loss [2,3,4,5,6,7], but adherence to the dietary prescription—regardless of diet composition—is the strongest predictor of weight loss [8,9]. While a multitude of factors are likely to influence adherence to dietary prescriptions for weight loss, changes in appetite [10], cravings [11], and diet satisfaction/acceptability [12] often occur during weight loss that likely contribute to poor long-term adherence to dietary prescriptions. Investigating and intervening on these factors may be a promising approach to improve adherence to dietary prescriptions and enhance the effectiveness of behavioral obesity treatment programs.

For example, lower hunger ratings were predictive of greater weight loss during lifestyle-only and lifestyle plus pharmacological interventions [10,13], and increased feelings of hunger following weight loss likely contribute to weight regain [14,15]. Data regarding food cravings while dieting are equivocal with reports of unchanged [16], increased [17], and decreased food cravings [15,18] during the weight loss program. Similarly, cravings for restricted foods while dieting were shown to be either increased [11] or decreased [19]. Increased momentary feelings of deprivation while dieting predicted lapses during behavioral weight loss [12], and an unwillingness to endure feelings of deprivation is a common reason for discontinuing dietary interventions [20,21]. Thus, interventions that reduce hunger, food cravings, and feelings of deprivation (e.g., increase or maintain diet satisfaction) during weight loss could promote greater diet adherence and improve weight loss outcomes.

Higher protein (HP) diets were shown to reduce hunger, increase satiety [22,23], reduce food cravings [19,24], and were better accepted compared to lower or normal protein diets [25,26,27]. These features of HP diets may enhance the long-term adoption of these diet patterns for weight management. Findings from systematic reviews and meta-analyses support modestly greater weight/fat loss and a retention of lean mass while consuming HP vs. lower or normal protein diets [4,7,28]. However, the impact of restricting and/or promoting specific protein foods (e.g., meat) within the context of HP energy-restricted diets on appetite, food cravings, and diet satisfaction has not been extensively studied.

The primary aim of the Beef WISE Study [29] was to compare changes in body weight, body composition, and indices of cardiometabolic health between two energy-restricted HP diets that included either a prescription to consume ≥4 weekly servings of lean beef (Beef, B) as the only source of red meat (i.e., beef, pork, veal, lamb, and mutton) or to consume no red meats (Non-Beef, NB) for 6 months. Body weight was reduced by 7.8 ± 5.9% in B and 7.7 ± 5.5% in NB, which indicated equivalent weight loss between the diets (mean difference: 0.06%, 90% confidence interval: (−1.7, 1.8)). Reductions in fat mass (B: 8.0 ± 0.6 kg and NB: 8.6 ± 0.6 kg) and improvements in markers of cardiometabolic health were not different between diets [29]. The current manuscript presents results from a secondary *a priori* aim of the Beef WISE Study, which was to determine if subjective appetite ratings (hunger and fullness), cravings, and diet satisfaction differed between B and NB during the weight loss intervention. It was hypothesized that hunger and fullness ratings would not differ between B and NB due to similar total prescribed protein intakes [22], but that NB would report greater cravings and lower diet satisfaction than B due to the restriction of all red meats (including beef) in the NB diet plan and the broad popularity of beef in the U.S. [30]. The broader relationships of appetite, cravings, and diet satisfaction with achieved weight loss independent of the group of assignment were also investigated. It was hypothesized that lower hunger, lower food cravings, and higher diet satisfaction would be associated with greater weight loss independent of the assigned group.

## 2. Materials and Methods

### 2.1. Participants

Adults with overweight/obesity were recruited from the Denver, CO metropolitan area to participate in a behavioral weight loss study at the University of Colorado Anschutz Health and Wellness Center (AHWC). The inclusion criteria for the study were as follows: Male or female; age 18–50 years; BMI ≥ 27.0 kg/m^2^; weight stable (±3 kg in previous 3 months); able to progress to 70 min/day of moderate intensity exercise (e.g., brisk walking); willing to comply with all study procedures including attendance to 16 weekly classes and 3 study visits. Individuals were excluded from the study for the following reasons: Pregnant or trying to become pregnant; diagnosis of diabetes; LDL cholesterol >160 mg/dl; triglycerides >400 mg/dl; untreated or unstable hypothyroidism; medication use that could cause weight loss or gain; following vegetarian or vegan diet; current eating disorder (e.g., anorexia, bulimia, binge eating disorder); any medical condition for which consuming a HP diet and/or engaging in 70 min of exercise daily would be inadvisable.

### 2.2. Experimental Design

Subjects were randomly assigned to one of two energy-restricted diets: A HP diet with instructions to consume ≥4 weekly servings of lean beef as the only source of red meat (B), or a HP diet with instructions not to consume any red meat for the duration of the study (NB). The details of the weight loss intervention were previously published [29]. Briefly, all subjects participated in the *State of Slim* (SOS) group-based weight management program [31] at the AHWC for 16 weeks. Following the 16-week SOS program, subjects were instructed to continue following the SOS diet plan and B or NB assignment for an additional 8 weeks. Subjects had no contact with the SOS group leader or research staff during this time (except to schedule and confirm the week 24 testing visit).

The SOS diet plan is HP, low in fat, and emphasizes non-starchy (e.g., vegetable) and whole-grain carbohydrates. The diet plan is structured into 3 phases that include phase-specific food choices from which participants can choose in pre-determined portion sizes. The exact macronutrient distribution of the SOS diet plan is variable among participants (depends on specific chosen foods) and the 3 diet phases. Estimated macronutrient distributions for the SOS diet plan have been calculated and previously reported to be 26–32% carbohydrate, 40–50% protein, and 24–28% fat [29]. Protein foods in the SOS program are lean and minimally processed (i.e., lean meat and poultry, fish, egg whites, and fat-free dairy) [31]. With the exception of lean beef in the B group, recommendations for protein sources and total protein intakes were the same between B and NB. The SOS program also emphasizes portion-control rather than counting calories. For this reason, and due to the known limitations of current methods of self-reported dietary intakes [32], self-reported energy intake and macronutrient distribution were not tracked during the Beef WISE Study.

Ratings of daily hunger, fullness, and cravings were measured for 7 days at the baseline, week 8, week 16, and week 24. Measures of diet satisfaction were completed at week 16 (end of the SOS group classes) and week 24. Body weight was measured at the baseline, week 16, and week 24. All subjects provided written informed consent and received a monetary stipend. The consent form and all study procedures and documents were approved for use by the Colorado Multiple Institutional Review Board. The study was registered on ClinicalTrials.gov (NCT02627105).

### 2.3. Appetite and Craving Ratings

Subjects rated their feelings of hunger, fullness, and food cravings on 100-mm visual analog scales (VAS) for 7 days at the baseline, week 8, week 16, and week 24. The VAS were anchored with “Not at all” and “Extremely,” which corresponded to scores of 0 and 100, respectively. Subjects were asked to complete the VAS at the end of each day to report their overall feelings of hunger, fullness, and cravings throughout the day. Subjects used a black or blue pen to make a vertical line (perpendicular to the 100-mm scale) to indicate their level hunger, fullness, and food cravings over the course of the day. Researchers used a ruler to measure the distance from 0 (“Not at all”) to the mark to calculate each daily rating. Daily ratings for each 7-day period were averaged to create a continuous numerical value to represent ratings for hunger, fullness, and food cravings at each time-point. VAS for hunger and fullness have been validated and used extensively in the literature [33]. The VAS for cravings was created by the research group using a similar format (e.g., 100-mm scale and anchors of “Not at all” and “Extremely”) as those for hunger and fullness and asked participants to rate their “overall food cravings” over the course of the day. The VAS for hunger, fullness, and food cravings are included in the Supplemental Materials.

### 2.4. Diet Satisfaction

Using a 5-point Likert scale (Not at All, Somewhat, Moderately, Very, Extremely), subjects rated their overall compliance with the diet plan, overall satisfaction with the diet plan, and feelings of deprivation on the diet plan at week 16 and week 24. Diet satisfaction measures were completed on a personal computer located in the AHWC Clinical Research Center using the REDCap data capture tool [34]. Research staff did not routinely provide subjects with additional information on how to complete the Likert scales, but they were available to provide assistance on an as-needed basis. The scales for compliance, satisfaction, and deprivation are included in the Supplemental Materials.

### 2.5. Anthropometric Measurements

A digital platform scale (PS-6600 ST, Befour, Inc., Saukville, WI, USA) was used to measure body weight in the AHWC clinical research center. Body weight was measured at the baseline, the end of the weight loss intervention (week 16), and at the end of the 8-week follow-up period (week 24). Subjects were weighed after an overnight fast, wearing light clothing, and after voiding. Height was measured using a stadiometer at the baseline. Body mass index (BMI; kg/m^2^) was calculated using these measurements.

### 2.6. Statistical Analysis

Study data were collected and managed using REDCap electronic data capture tools hosted at the University of Colorado Anschutz Medical Campus. REDCap (Research Electronic Data Capture) is a secure, web-based application designed to support data capture for research studies, providing the following: (1) An intuitive interface for validated data entry; (2) audit trails for tracking data manipulation and export procedures; (3) automated export procedures for seamless data downloads to common statistical packages; and (4) procedures for importing data from external sources [34].

Statistical power calculations were not completed for the analyses presented in the current manuscript, which were secondary aims of the Beef WISE Study. Power calculations for the Beef WISE Study were completed for the primary aim of comparing weight loss between B and NB. Baseline age, body weight, and BMI were calculated and reported as mean ± SD. Detailed baseline demographic, clinical, and lab data were published previously [29] and are not reported here. Linear mixed effects models with compound symmetry covariance were used to test for effects of time, group (B vs. NB), and their interaction term on hunger, fullness, and craving rating. Diet compliance, satisfaction, and deprivation at week 16 and 24 were compared between B and NP using Student’s *t*-tests for two independent samples. Results from the linear mixed model analyses (hunger, fullness, and cravings) are reported as LSMEANS ± SE. Results from the independent *t*-tests (compliance, satisfaction, and deprivation) are reported as mean ± SD. Statistical significance was indicated at α = 0.05.

Pearson’s correlation coefficients were used to determine linear relationships between the baseline and week 16 and 24 hunger, fullness, and craving VAS scores and weight loss at week 16 and week 24. Linear relationships were also assessed between diet compliance, satisfaction, and deprivation ratings at week 16 and weight loss at week 16 and week 24. These analyses were completed without consideration of group assignment in the study to investigate the broader relationships among appetite and diet satisfaction variables and weight loss. Results are reported as Pearson’s correlation coefficients (*r*) and *α* = 0.05 were used to determine statistical significance.

## 3. Results

### 3.1. Participant Characteristics

**Figure 1:**
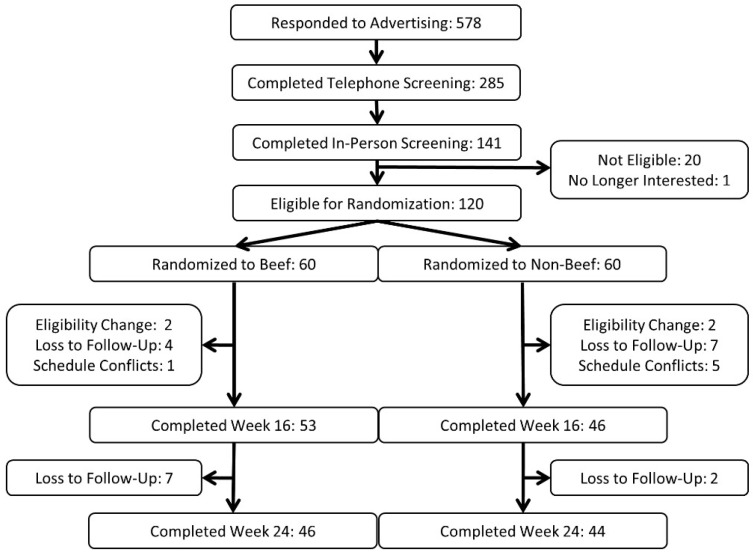
Study recruitment and flow diagram.

One-hundred twenty individuals (99 female, 21 male) enrolled in the study. Ninety-nine individuals (83 female, 16 male, 82.5%) completed the group-based weight loss phase of the study (16 weeks) and 90 individuals (76 female, 14 male, 75%) completed the entire six-month study (Figure 1). Detailed participant characteristics were reported previously and indicated a healthy, obese study population (indices of cardiometabolic health were within normal reference ranges) [29]. Abbreviated baseline characteristics are presented in Table 1. Those assigned to B (36.0 ± 8.3 years) were younger than NB (39.3 ± 7.8 years, *p* = 0.026). The addition of age as a covariate to statistical models did not influence the results.

### 3.2. Appetite and Cravings

**Figure 2:**
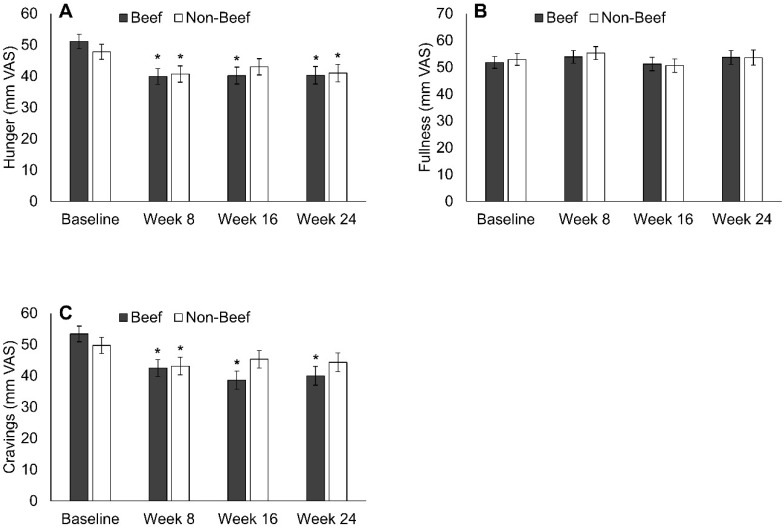
Changes in hunger (**A**), fullness (**B**), and cravings (**C**) during the Beef WISE Study. Hunger was reduced in both groups at weeks 8 and 24 compared to baseline, and also at week 16 in B. Fullness during weight loss was not different from baseline. Cravings were reduced in B at all intervention time points compared to baseline, and were reduced in NB at week 8 but not weeks 16 or 24. * Indicates significantly different from baseline (*p* < 0.05) by linear mixed model analyses (SAS, Proc Mixed).

Self-reported daily hunger was reduced in B at weeks 8, 16, and 24 compared to the baseline and in NB at weeks 8 and 24 (Figure 2, Supplemental Table S1). Daily fullness was unaltered during the weight loss intervention compared to the baseline (Figure 2, Supplemental Table S1). Compared to the baseline, daily overall cravings were reduced at weeks 8, 16, and 24 in B and at week 8 in NB (Figure 2, Supplemental Table S1). The overall time by group interactions for hunger (*p* = 0.47), fullness (*p* = 0.96), and craving (*p* = 0.096) were not significant. The absolute hunger, fullness, and craving ratings were not different between B and NB at any time point, but the change in cravings from the baseline to week 16 was significantly greater in B vs. NB (significant time by group interaction at week 16, *p* = 0.018). Across all participants, the baseline hunger and craving VAS scores were linearly and inversely associated with weight loss at week 16 (hunger: *r* = −0.22, *p* = 0.031, craving: *r* = −0.20, *p* = 0.048) and week 24 (hunger: *r* = −0.29, *p* = 0.007, craving: *r* = −0.23, *p* = 0.037, Table 2). The hunger and craving scores at weeks 16 and 24 were not associated with weight loss, except for hunger at week 24 with weight loss at week 24 (*r* = −0.25, *p* = 0.033, Table 2). Fullness ratings were not associated with changes in body weight at any time point (Table 2).

### 3.3. Diet Satisfaction and Compliance

**Figure 3:**
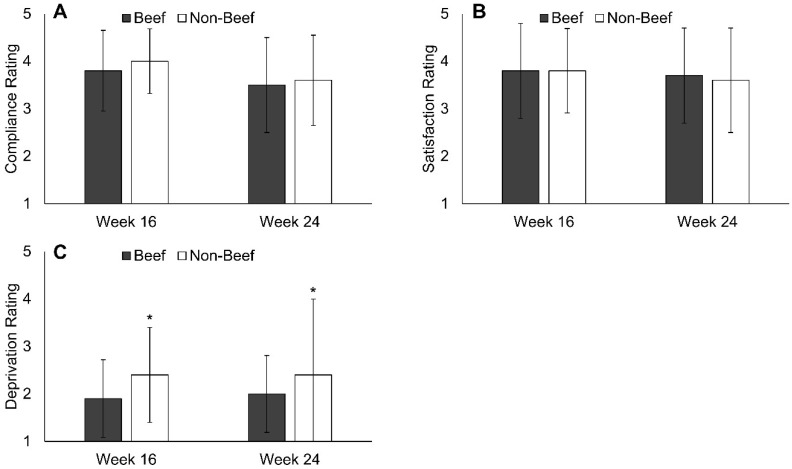
Ratings of dietary compliance (**A**) and satisfaction (**B**) were not different between groups during the Beef WISE Study. Feelings of deprivation (**C**) were greater in NB vs. B at weeks 16 and 24. * Indicates significant difference between groups (*p* < 0.05) by independent t-tests (SAS, Proc Ttest).

Overall, participants reported high diet satisfaction and compliance with the B and NB diet plans with no difference between groups (Figure 3, Supplemental Table S2). Feelings of deprivation were higher in NB vs. B at week 16 and week 24 (Figure 3, Supplemental Table S2). Across all subjects, self-reported compliance and diet satisfaction were linearly and positively associated with weight loss at week 16 (compliance: *r* = 0.52, *p* < 0.001, satisfaction: *r* = 0.33, *p* = 0.001) and week 24 (compliance: *r* = 0.52, *p* < 0.001, satisfaction: *r* = 0.32, *p* = 0.002, Table 3). Self-reported feelings of deprivation were linearly and inversely associated with weight loss at week 16 (*r* = −0.21, *p* = 0.043) and week 24 (*r* = −0.24, *p* = 0.028, Table 3).

## 4. Discussion

Primary results of the Beef WISE Study were published previously [29], and a secondary aim of the study was to investigate potential differences in appetite and diet satisfaction between two HP diets that differed in red meat (specifically lean beef) intakes. It was hypothesized (*a priori*) that both diets would similarly reduce hunger and increase fullness, butthat the HP diet with beef would promote greater diet satisfaction and compliance. Results of this current secondary analysis of the Beef WISE Study support some, but not all, of those hypotheses. As hypothesized and consistent with HP diets, feelings of daily hunger were reduced compared to the baseline in both groups, but daily fullness was not different from the baseline. The observed reduction in daily hunger with no effect on daily fullness/satiety is notable because HP meals and diets are generally thought to have stronger effects on enhancing postprandial satiety than on reducing hunger [22]. Feelings of deprivation were higher in NB compared to B as hypothesized, but the overall deprivation ratings were relatively low in both groups (2 = “Somewhat Deprived”) and highly variable. Contrary to the a priori hypotheses, cravings, dietary compliance, and diet satisfaction were not different between B and NB with the exception of a greater reduction in cravings at week 16 in B vs. NB. However, these results are broadly consistent with the overall findings of the Beef WISE Study that demonstrated equivalent weight loss and no difference in changes of body composition or cardiometabolic health between B and NB [29].

A second aim of the current analysis of the Beef WISE Study was to investigate the broader relationships of self-reported appetite and diet satisfaction with weight loss, independent of the group assignment in the trial. Changes in body weight are highly variable during behavioral weight loss interventions, and maintaining weight loss after the intervention is especially difficult for most individuals [9,35]. Recent initiatives, such as the NIH-supported ADOPT (Accumulating Data to Optimally Predict Obesity Treatment) Core Measures working group [36], have increased the emphasis on investigating the underlying reasons for the high inter-individual variability in weight loss and poor long-term weight loss outcomes with behavioral weight loss interventions. Self-reported appetite and craving ratings prior to initiating obesity treatment are a potentially important moderator of weight loss success [10,13,14,15], and to help understand the variability in response to obesity treatment, the ADOPT Psychosocial Domain Subgroup identified hunger, satiety (fullness), and cravings as high priority constructs to be measured during behavioral weight loss [37]. In the current study, greater baseline hunger and craving ratings were associated with less weight loss during the intervention, but hunger and craving ratings during the intervention were mostly not associated with weight loss. Only hunger ratings at week 24 were significantly correlated with week 24 weight loss. 

These finding are important for two reasons. First, the ability to easily identify individuals with high baseline hunger and cravings represents a potentially important intervention target for behavioral weight loss. For example, those with higher baseline hunger might receive more intensive counseling or be good candidates for using weight loss medications (such as phentermine) that help patients manage hunger and improve weight loss outcomes [13]. Second, increased hunger during weight loss is a commonly-cited factor for explaining poor weight loss outcomes during behavioral interventions [37]. Results from the current study do not, however, support the commonly-held notion of hunger and cravings during weight loss as important predictors of success, at least during a 6-month intervention with HP diets with or without beef. It is possible that hunger and cravings while dieting may become more important for moderating success with weight loss maintenance rather than active weight loss, which is consistent with the current finding of the relationship between hunger at the conclusion of the study (week 24) and weight loss at week 24. These findings are also consistent with weight loss and weight loss maintenance being distinct physiological and psychological states that likely require different treatment approaches [38].

Results from the current study also support past research demonstrating that adherence to a dietary prescription is the single most important predictor of weight loss [8,9]. The average self-reported compliance to the dietary prescription was relatively high at weeks 16 and 24 (3.7 out of 5 corresponds to between “moderately” and “very” compliant), and the degree of reported compliance was the strongest predictor of weight loss in the current study, which explained approximately 25% of the variability in observed weight loss. That the most compliant subjects achieved the greatest weight loss is not surprising, but the implications of the finding are important for future research and behavioral weight loss programs. The vast majority of past research on obesity treatment—including the Beef WISE Study—has focused on the comparative effectiveness of one weight loss strategy vs. another (e.g., varying macronutrient composition of the diet, with vs. without exercise, etc.), but this approach has done little to improve long-term weight loss outcomes and has caused substantial confusion and distrust among the general populous regarding effective behavioral weight management strategies. Future research should place less emphasis on discovering the “silver bullet” diet and/or exercise prescription for weight loss, and shift the focus to strategies that increase adherence to the prescription, regardless of the details of the prescription. Such a focus also requires a deeper understanding of the numerous biological, behavioral, environmental, and psychosocial factors that likely moderate the success of weight loss interventions [9,36,39], and it requires tailoring specific weight loss and weight loss maintenance strategies to address those individualized factors.

Investigating the influence of behavioral/psychosocial factors at the baseline and during the intervention on weight loss outcomes has been recommended by the ADOPT Core Measures Working Group [36,37] and the 2016–2021 National Nutrition Research Roadmap [39], which represents a significant strength of the current study. The parent intervention trial (Beef WISE Study [29]) was based on a comprehensive behavioral weight management program (*State of Slim* [31]) that is consistent with published guidelines for the treatment of obesity [1] and is available to the general public through purchase of the book and/or enrollment in the commercial, fee-based program. These features represent another strength of the current research because the current findings can be translated and implemented in the SOS program to potentially improve weight loss outcomes in pragmatic settings.

There are some limitations of the current study that deserve mention and consideration for future research. The results of the current research were secondary aims of the Beef WISE Study, and statistical power calculations were completed for these aims. The Beef WISE Study was limited by the lack of a lower protein control group (protein intake near the RDA of 0.8 g/kg body weight), which would have allowed for more definitive analyses of the impact of diet-specific effects on appetite, cravings, and diet satisfaction and their impacts on weight loss. Ratings of hunger, fullness, and cravings were collected on a daily basis (overall feelings of hunger, fullness, and cravings throughout the day) for 7 days at weeks 8, 16, and 24. Collecting these data at multiple times throughout the day (for example hourly or before/after meals) would have provided a more complete understanding of how these factors changed during the trial and influenced weight loss, but that would have added a substantial burden to both subjects and researchers. Subjects were asked to rate their non-specific or overall cravings throughout the day during the Beef WISE Study. The only difference in dietary prescriptions between B and NB was related to the consumption of red meats. Asking participants about their cravings for beef and/or red meats may have provided additional information regarding the impact of cravings specifically for restricted foods on weight loss. The craving VAS was created by the research team and was therefore not validated prior to its use in the Beef WISE Study. However, the scale was created with a similar format as VAS for appetite (e.g., 100-mm scale and anchors of “Not at all” and “Extremely”), which has been used extensively in human clinical research [33]. Lastly, the Beef WISE Study is limited by a short follow-up duration of 8 weeks after the conclusion of the 16-week group-based weight loss intervention. A longer follow-up period would have allowed for the investigation of whether observed differences in deprivation and craving scores between groups persisted and/or influenced long-term weight loss maintenance.

## 5. Conclusions

In conclusion, self-reported measures of appetite, diet compliance, and diet satisfaction were not different between two high-protein diets that differed in the amount of lean beef consumed. Subjects assigned to a high-protein diet with ≥4 weekly servings of lean beef reported lower feelings of deprivation and a greater reduction in cravings compared to a high-protein diet restricted in all red meats. However, these potential benefits of a high-protein diet with lean beef did not lead to greater weight loss. Baseline appetitive factors (hunger and cravings) and diet satisfaction during the intervention (compliance, satisfaction, and deprivation) were associated with 16- and 24-week weight loss independent of diet assignment. Collectively, these findings suggest that future research should focus on developing behavioral weight loss strategies that promote reduced feelings of hunger, cravings, and deprivation to improve adherence to dietary prescriptions and improve long-term weight loss outcomes.

## Figures and Tables

**Table 1:** Baseline participant characteristics ^1^.

Parameter	All	Beef	Non-Beef
Sample Size (# Female)	120 (99)	60 (49)	60 (50)
Age (year)	37.6 ± 8.1	36.0 ± 8.3	39.3 ± 7.8 *
Body Weight (kg)	101.1 ± 22.8	100.8 ± 21.9	101.5 ± 24.0
BMI (kg/m^2^)	35.7 ± 7.0	35.9 ± 6.8	35.4 ± 7.1

^1^ Data are presented as mean ± standard deviation. * Indicates significant difference (*p* < 0.05) between Beef and Non-Beef by unpaired *t*-test (SAS, Proc Ttest). BMI, body mass index; #, number.

**Table 2:** Correlations of hunger, fullness, and food cravings with weight loss.

Parameter	Pearson *r* for Week 16 Weight Loss	*p* Value for Week 16 Weight Loss	Pearson *r* for Week 24 Weight Loss	*p* Value for Week 24 Weight Loss
Hunger				
Baseline	−0.22 *	0.031	−0.29 *	0.007
Week 16	−0.13	0.233	−0.17	0.123
Week 24	--	--	−0.25 *	0.033
Fullness				
Baseline	−0.11	0.280	−0.13	0.250
Week 16	−0.17	0.132	−0.06	0.627
Week 24	--	--	−0.06	0.586
Food Cravings				
Baseline	−0.20 *	0.048	−0.23 *	0.037
Week 16	−0.08	0.497	−0.11	0.312
Week 24	--	--	−0.14	0.228

* Indicates significant linear correlations (*p* < 0.05) by Pearson *r* (SAS, Proc Corr).

**Table 3:** Correlations of diet compliance, diet satisfaction, and deprivation with weight loss.

Parameter	Pearson *r* for Week 16 Weight Loss	*p* Value for Week 16 Weight Loss	Pearson *r* for Week 24 Weight Loss	*p* Value for Week 24 Weight Loss
Compliance	0.52 *	<0.001	0.52 *	<0.001
Satisfaction	0.33 *	0.001	0.32 *	0.002
Deprivation	−0.21 *	0.043	−0.24 *	0.028

* Indicates significant linear correlations (*p* < 0.05) by Pearson *r* (SAS, Proc Corr).

## Supplementary Materials

The following are available online at http://www.mdpi.com/2072-6643/10/6/700/s1, Table S1: Reported Appetite and Craving Visual Analog Scale Scores during Weight Loss, Table S2: Self-Reported Compliance, Satisfaction, and Deprivation with the Diet Plan.
