# Supplementary material for: Hunger, Food Cravings, and Diet Satisfaction are Related to Changes in Body Weight During a 6-Month Behavioral Weight Loss Intervention: The Beef WISE Study
Source: Nutrients. 2018 May 31;10(6):700. doi: 10.3390/nu10060700 (PMC6024699; doi:10.3390/nu10060700)
Supplement: Supplementary file 1 [file nutrients-10-00700-s001.pdf]

Supplemental Table 1: Reported Appetite and Craving Visual Analog Scale Scores during Weight Loss[1].

Parameter	Group	Baseline	Week 8	Week 16	Week 24
**Hunger**	Beef	51.1 ± 2.3	39.9 ± 2.5*	40.2 ± 2.7*	40.3 ± 2.8*
	Non-Beef	47.8 ± 2.4	40.7 ± 2.6*	43.0 ± 2.6	41.0 ± 2.8*
	Difference[2]	3.4 ± 3.3	-0.8 ± 3.6	-2.9 ± 3.7	-0.7 ± 4.0
**Fullness**	Beef	51.8 ± 2.2	53.9 ± 2.4	51.2 ± 2.5	53.7 ± 2.6
	Non-Beef	52.9 ± 2.2	55.3 ± 2.4	50.6 ± 2.5	53.6 ± 2.8
	Difference	-1.0 ± 3.1	-1.4 ± 3.4	0.6 ± 3.5	0.03 ± 3.7
**Cravings**	Beef	53.4 ± 2.5	42.5 ± 2.7*	38.6 ± 2.9*	40.0 ± 3.0*
	Non-Beef	49.7 ± 2.6	43.1 ± 2.8*	45.3 ± 2.8	44.3 ± 3.0
	Difference	3.7 ± 3.6	-0.7 ± 3.9	-6.8 ± 4.0	-4.3 ± 4.3

[1] Values are LSMEANS ± SE from the linear mixed effects model.

[2] Between group differences calculated as Beef – Non-Beef. Differences may not exactly reflect values for Beef and Non-Beef due to rounding.

A linear mixed effects model (SAS, Proc Mixed) was used to compare hunger, fullness, and craving ratings at baseline, week 8, week 16, and week 24 between Beef and Non-Beef groups. * indicates a within-group difference in rating at time point vs. baseline ($p < 0.05$).

Supplemental Table 2: Self-Reported Compliance, Satisfaction, and Deprivation with the Diet Plan[1].

Parameter	Group	Week 16	Week 24
**Compliance**	Beef	3.8 ± 0.85	3.5 ± 1.0
	Non-Beef	4.0 ± 0.68	3.6 ± 0.95
	Difference[2]	-0.16 ± 0.78	-0.11 ± 1.0
**Satisfaction**	Beef	3.8 ± 1.0	3.7 ± 1.0
	Non-Beef	3.8 ± 0.89	3.6 ± 1.1
	Difference	-0.09 ± 0.95	0.11 ± 1.1
**Deprivation**	Beef	1.9 ± 0.82	2.0 ± 0.81
	Non-Beef	2.4 ± 1.0	2.4 ± 1.6
	Difference	-0.48 ± 0.94*	-0.44 ± 1.0*

[1] Values are Mean ± SD.

[2] Between group differences calculated as Beef – Non-Beef. Differences may not exactly reflect values for Beef and Non-Beef due to rounding.

A linear mixed effects model (SAS, Proc Mixed) was used to compare compliance, satisfaction, and deprivation ratings at week 16 and week 24 between Beef and Non-Beef groups. Ratings were completed using a Likert Scale with ratings from 1 (Not at All) to 5 (Extremely). * indicates a significant ($p < 0.05$) between-group difference.
